# Molecular Research on Reproductive Toxicity

**DOI:** 10.3390/ijms24043538

**Published:** 2023-02-10

**Authors:** Rosaria Scudiero

**Affiliations:** Department of Biology, University Federico II, Via Cintia 21, 80126 Napoli, Italy; rosaria.scudiero@unina.it

Fertility rates in animals have shown a progressive decrease in recent decades, and reproductive toxicity is considered an important regulatory endpoint in health hazard assessment. The focus of the Special Issue “Molecular Research in Reproductive Toxicology” is to highlight the benefits of experimental approaches in elucidating the cellular mechanisms and molecular players involved in the failures of reproductive processes, such as gametogenesis, fertilization, mammalian blastocyst implantation and embryogenesis. The variety of animal models used, from sea urchins to fish, lizards and mammals, contributes to the knowledge of the fundamental reproductive processes and of the strategies to be implemented in defense of animal reproduction and broadens the audience to which this Special Issue can be addressed. Ten published manuscripts, including seven research articles and three reviews, can be found in this Special Issue. Two articles [1,2] underline the importance of the redox balance in sperm functions. Mottola et al. [1] report for the first time the antigenotoxic effects of the combined exposure to ascorbic and ellagic acids, two well-known antioxidants, on human spermatozoa in vitro. The authors demonstrated that the combination of the two antioxidants generates a time-dependent antigenotoxic action against benzene, reducing both the sperm DNA fragmentation index and oxidative stress, putting the foundations for clinical studies on the use of these class of antioxidants as a therapy for male infertility. In the second article of Soria-Tiedemann et al. [2], starting from the information that Arachidonate 15-lipoxygenase (Alox15) and Glutathione peroxidase 4 (Gpx4) enzymes are both involved in sperm maturation, the authors explored the potential roles of these enzymes in acrosomal exocytosis and in vitro fertilization. Using mice carrying genetic alterations, they found that Gpx4 is abundantly present in the sperm head and that its subcellular distribution is altered during sperm capacitation, in fact sperm from mice with a silent Gpx4 gene exhibit a reduced ability of acrosomal exocytosis. Active Gpx4 in sperm is essential for in vitro fertilization, whereas the presence and absence of Alox15 does not play a major role.

Two other articles published in this Special Issue [3,4] focus on in vitro oocyte maturation and implantation in mammals. In particular, Park et al. [3] described how porcine oocytes benefit from seven passages in equine amniotic fluid mesenchymal stem cell conditioned medium (eAFMSC-CM); the treatment generates a high antioxidant activity in the oocytes, enhancing their maturation and subsequent embryonic development. The antioxidant effect of the eAFMSC-CM can regulate the expression of pluripotency and apoptosis genes and is able to decrease autophagy in blastocysts. The research by Neuper and coworkers [4] investigated the possible role of candesartan on trophoblastic peroxisome proliferator-activated receptor gamma (PPARγ) and its hallmark target genes in early gestation. Candesartan is an angiotensin II receptor 1 blocker commonly used to treat hypertension and acts as a PPARγ agonist, which, in turn, is a key regulator important for placenta development. Using human cytotrophoblast/syncytiotrophoblast cell models and placental explants, the authors demonstrated that candesartan does not affect the PPARγ protein expression or its nuclear translocation. However, by profiling the target–receptor expression using single-cell RNA-sequencing, they demonstrated that early gestational myofibroblasts express the candesartan target–receptor angiotensin II type 1 receptor (AGTR1) causing adverse effects, including impaired placental angiogenesis. 

The other five papers published in the Special Issue “Molecular Research in Reproductive Toxicology” have environmental changes as the main topic, mainly given by environmental pollution from emerging contaminants and the damage that it can cause regarding reproductive processes. Among environmental changes, global warming/cooling represents one of the main challenges for animals; aquatic organisms are currently the most exposed to unexpected changes in water temperature. Temperature is known to control fish development and reproduction. In the study by Ge et al. [5], the effect of a drop in temperature on zebrafish spawning was investigated and key genes, hub pathways and important biological processes closely associated with female spawning were identified through the transcriptomic analysis of zebrafish brain tissue. The authors demonstrated that temperature at 19 °C did not influence zebrafish fecundity but a temperature of <19 °C significantly blocked spawning, suggesting the existence of a low temperature critical point for the spawning. Both downregulated and upregulated genes were found during the temperature reduction. Analysis indicated that the most important biological processes inhibited at temperatures of <19 °C included the GnRH signaling pathway, vascular smooth muscle contraction, C-type lectin receptor signaling pathway, phosphatidylinositol signaling system, the organization and the maintenance of the photoreceptor cell outer segment, the circadian regulation of gene expression and the calcium signaling and insulin signaling pathways. These findings uncovered crucial hormone-related genes and signaling pathways controlling the spawning of female zebrafish under cold stress.

Using sea urchins as an experimental model, two articles explored the effect of certain metals on gametogenesis and embryonic development. In particular, the review by Martino et al. [6] focuses on the impact of rare earth elements (REEs), also known as lanthanides, whose increasing presence in the environment has drawn the attention of the scientific community regarding their safety and toxicity. The multiple sources of REEs in the environment range from diagnostic medicine to various industries; their exponential use and the poor management of waste disposal raise serious concerns about the quality and safety of the aquatic ecosystems. Findings collected on sea urchin gametes and embryos and illustrated in the review demonstrated that REE exposure triggers a wide variety of toxic insults, including reproductive performance, fertilization, redox metabolism, embryogenesis and the regulation of embryonic gene expression. The uptake of gadolinium, the most widely used lanthanide in diagnostic medicine, in sea urchin embryos occurs in a time- and concentration-dependent manner and correlates with reduced calcium uptake, mainly affecting skeletal growth and resulting in the dysregulation of the skeletal gene regulatory network. Scientific evidence also shows variable sensitivity of the early life stages of different species, highlighting the importance of testing the effects of pollution in different species. Another paper featuring sea urchin embryos is a research article by Chiarelli and coworkers [7] which studied the toxicity of vanadium (V). V is widely used in industrial and biomedical fields and represents an emerging environmental pollutant because wastewater treatment plants do not adequately remove its compounds. The article describes how V perturbs sea urchin embryogenesis and skeletogenesis, triggering several stress responses: the metal interferes with calcium uptake, causes a disruption in the biomineralization process, modulates the ERK pathway and activates a cell-selective apoptosis. Results once again endorse the sea urchin embryo as an adequate experimental model to study metal-related cellular/molecular responses.

The expansion of intensive agricultural practices, aimed at maximizing crop yields, has led to a sharp increase in the contamination of water and soil by pesticides and herbicides, substances known to have serious negative effects on reproductive processes. The review of Terayama et al. [8] highlights on the effect of the neonicotinoids (NP), neurotoxic substances highly effective as pesticides owing to their water solubility, permeability and long-lasting activity. In particular, the review summarizes recent evidence on NP-induced testicular and ovarian toxicity, which revealed that the mechanism of injury is mainly driven by oxidative stress. The authors also reported the seasonal variations in NP concentrations found in river systems in Japanese regions, where NP use is not declining, unlike worldwide.

Finally, two articles published in this *IJMS* Special Issue [9,10] have an unconventional animal model as their subject, but which can be easily exposed in nature to environmental contamination, i.e., the field lizard *Podarcis siculus*. The first illustrates the adverse effects of glyphosate (Gly) on lizard testis. Taking advantage of previous data demonstrating the high toxicity of this herbicide on the liver of these animals [11], the authors evaluated testicular structure, steroidogenesis and estrogen receptor expression/localization after oral exposure to Gly. The results show that Gly affected testicular morphology, reduced spermatogenesis, altered gap junctions and changed the localization of estrogen receptors in germ cells, indicating that this herbicide can disturb the morpho-physiology of male reproductive system, with obviously harmful effects on the reproductive fitness of lizards.

The last article [10] is a review summarizing the available data on the effects of different environmental pollutants on the control of spermatogenesis in the lizard *P. siculus*. Data show that any exogenous substance capable of dysregulating the production of factors controlling spermatogenesis leads to the structural and functional alterations of the testis: changes in the levels of sex hormones, pituitary gonadotropins and androgen and estrogen receptors; the inhibition of aromatase enzyme; the thinning of seminiferous tubules epithelium; and the loss of connections between germ cells and Sertoli cells. The major morphological damage found in the seminiferous tubules of differently exposed males was a drop in spermatids and spermatozoa, demonstrating that the latter are the most sensitive stages of germ cell differentiation. If, on the one hand, this result indicates that altered levels of endogenous or exogenous substances lead to infertility due to a lack of spermatozoa, on the other, it demonstrates that the interruption of these perturbative effects could restore the efficiency of the seminiferous tubules and the production of spermatozoa, as they do not irreversibly affect spermatogonial stem cells.

In conclusion, data provided and reviewed in this Special Issue indicate the importance of antioxidants in the formation and maintenance of healthy gametes, the action of emerging environmental contaminants in promoting morphological and functional disorders in gonads, as well as teratogenesis. As Guest Editor, I hope that the themes and areas covered in this Special Issue will attract readers from the wider scientific community, help to expand our understanding on reproductive biology and enhance and strengthen strategies to be implemented in defense of the reproductive success. Finally, I would like to gratefully acknowledge and credit the contributions of all authors of both the original research articles and review papers on this relevant and valuable research topic.
